# Improvement of the Stability of Quantum-Dot Light Emitting Diodes Using Inorganic HfO_x_ Hole Transport Layer

**DOI:** 10.3390/ma17194739

**Published:** 2024-09-27

**Authors:** Jung Min Yun, Min Ho Park, Yu Bin Kim, Min Jung Choi, Seunghwan Kim, Yeonjin Yi, Soohyung Park, Seong Jun Kang

**Affiliations:** 1 Department of Advanced Materials Engineering for Information and Electronics, Kyung Hee University, Yongin 17104, Republic of Korea; msd426@khu.ac.kr (J.M.Y.); triolight@khu.ac.kr (M.H.P.); bebeto@khu.ac.kr (Y.B.K.); okcmj@khu.ac.kr (M.J.C.); 2 Integrated Education Program for Frontier Materials (BK21 Four), Kyung Hee University, Yongin 17104, Republic of Korea; 3 Advanced Analysis and Data Center, Korea Institute of Science and Technology (KIST), Seoul 02792, Republic of Korea; clam1127@kist.re.kr (S.K.); soohyung.park@kist.re.kr (S.P.); 4 Department of Physics, Yonsei University, Seoul 03722, Republic of Korea; yeonjin@yonsei.ac.kr; 5 Division of Nanoscience & Technology, KIST School, University of Science and Technology (UST), Seoul 02792, Republic of Korea

**Keywords:** QLEDs, all-inorganic device, quantum dots, stable, oxygen vacancies, optoelectronics, solution process

## Abstract

One of the major challenges in QLED research is improving the stability of the devices. In this study, we fabricated all inorganic quantum-dot light emitting diodes (QLEDs) using hafnium oxide (HfO_x_) as the hole transport layer (HTL), a material commonly used for insulator. Oxygen vacancies in HfO_x_ create defect states below the Fermi level, providing a pathway for hole injection. The concentration of these oxygen vacancies can be controlled by the annealing temperature. We optimized the all-inorganic QLEDs with HfO_x_ as the HTL by changing the annealing temperature. The optimized QLEDs with HfO_x_ as the HTL showed a maximum luminance and current efficiency of 66,258 cd/m^2^ and 9.7 cd/A, respectively. The fabricated all-inorganic QLEDs exhibited remarkable stability, particularly when compared to devices using organic materials for the HTL. Under extended storage in ambient conditions, the all-inorganic device demonstrated a significantly enhanced operating lifetime (T_50_) of 5.5 h, which is 11 times longer than that of QLEDs using an organic HTL. These results indicate that the all-inorganic QLEDs structure, with ITO/MoO_3_/HfO_x_/QDs/ZnMgO/Al, exhibits superior stability compared to organic-inorganic hybrid QLEDs.

## 1. Introduction

Colloidal quantum dot (QD) light-emitting diodes have been extensively researched over the past few decades as a next-generation display technology. Unlike other LEDs that use two-dimensional (2D) materials, organics, or perovskites in the emitting layer (EML), QLEDs that use QDs offer advantages such as easy band gap tuning for color customization, high stability, brightness, high color purity, and low cost fabrication [1,2,3,4,5,6].

In QLED research, significant efforts have been made not only to enhance emission performance, such as luminance and efficiency, but also to improve device stability. Factors contributing to improved QLED stability include charge balance optimization, QD ligand exchange, and layer modification [4,7,8]. Among these factors, layer modification arises from the weakness of organic materials to moisture and oxygen. This has led to extensive research on all-inorganic QLEDs [9,10,11,12]. A review of the research done so far indicates that various metal oxides, such as V_2_O_5_, MoO_3_, and WO_3_, are used as hole injection layers (HIL), while ZnO, ZnMgO, and SnO_2_ are commonly employed as electron transport layers (ETL), with these materials being optimized for solution processing [7,13,14,15,16,17].

However, the inorganic materials used for the hole transport layer (HTL) are limited, and films are fabricated through complex processes. For example, commonly used inorganic HTLs such as NiO and Al_2_O_3_ films are typically produced via physical deposition methods, and doping processes are often employed to enhance performance. In some cases, additional layers are inserted or further film engineering is performed to improve hole injection and optimize the device [11,14,18,19,20,21].

In this study, we fabricated all-inorganic QLEDs using HfO_x_ thin films, produced via solution processing, as the hole transport layer (HTL). Although HfO_x_ is widely used as an insulating layer and is not typically used as a charge transport layer (CTL) in LEDs, we used the defect states generated by oxygen vacancies within the HfO_x_ film. These defect states form below the Fermi level, and by controlling the annealing temperature after spin coating, we optimized the defect states to suit the intended device [22,23,24]. The optimized device achieved a maximum luminance of 66,258 cd/m^2^, a current efficiency of 9.7 cd/A, and an external quantum efficiency (EQE) of 2.4%. Additionally, the device exhibited an 11-fold increase in lifetime compared to a device using TFB as the HTL, along with superior stability when stored in ambient conditions. These findings demonstrate that solution-processed HfO_x_ HTL films can be used to create highly stable all-inorganic QLEDs.

## 2. Materials and Method

### 2.1. Solution Synthesis

The HfO_x_ solution was prepared by dissolving 0.01 M of HfCl_4_ (Sigma Aldrich, St. Louis, MO, USA) in ethylene glycol monomethyl ether. The solution was stirred for 1 h at room temperature. The MoO_3_ solution was prepared by dissolving 0.05 wt% of MoO_3_ nanoparticle powder (Sigma Aldrich, St. Louis, MO, USA) in deionized water. The solution of CdSe/ZnS Qds (Finelab, Deajeon, Republic of Korea) was prepared by diluting it in a toluene solution to a concentration of 20 mg/mL. The 15% Mg doped ZnMgO solution (Nanofix, Suwon, Republic of Korea) was prepared by diluting it in ethyl alcohol solution at a 1:1 ratio.

### 2.2. Fabrication of the Device

The patterned ITO glass was ultrasonicated with deionized water (DI water), acetone, and isopropyl alcohol (IPA) for 15 min, respectively. Subsequently, the substate was exposed to UV-Ozone for 15 min to make the surface hydrophilic, to remove residual organics, and to increase the work function, followed by four cycles of spin coating and annealing. First, the MoO_3_ solution was spin-coated on the substrates at 3000 rpm for 30 s, followed by annealing at 120 °C for 10 min. Next, for the all-inorganic QLEDs, the HfO_x_ solution was spin-coated at 3000 rpm for 30 s, followed by pre-annealing at 120 °C for 5 min to evaporate the solvent. Subsequently, three different devices were annealing at 150 °C, 250 °C, and 350 °C, respectively, for 45 min each. For the control device, a solution of TFB was spin-coated at 3000 rpm for 30 s and annealed at 180 °C for 30 min. Then, CdSe/ZnS QDs was spin coated onto the HTL layer at 2000 rpm for 30 s and annealed at 90 °C for 10 min. The optical band gap of the QDs was determined from UV-Vis measurements, and the photoluminescence (PL) spectra are shown in Figure S2a,b. The QDs layer was covered with a ZnMgO layer, which was spin-coated at 2000 rpm for 60 s and annealed at 90 °C for 10 min. Finally, a 130 nm aluminium cathode was deposited via thermal evaporation at a rate of 3 Å/s under a pressure of 8 × 10^−6^ torr.

### 2.3. Characterization and Measurement of Films and Devices

The absorption spectra were measured using UV-Vis spectroscopy (V-570, JASCO, Easton, MD, USA). The PL characteristics were measured with a PL spectrometer (Ocean Optics, USB 2000+, Rochester, NY, USA) using a 365 nm light source. The EL characteristics of the QLEDs were measured with a OLED I-V-L test system (M6100, Mcscience Inc., Suwon, Republic of Korea). The device structure was analyzed using TEM (ThermoFisher Talos, Waltham, MA, USA). Interfacial properties were investigated through XPS and UPS measurements (ThermoFisher NEXSA, Waltham, MA, USA) using Al Kα (1486.8 eV) and He–I (21.22 eV) sources.

## 3. Results and Discussion

**Figure 1:**
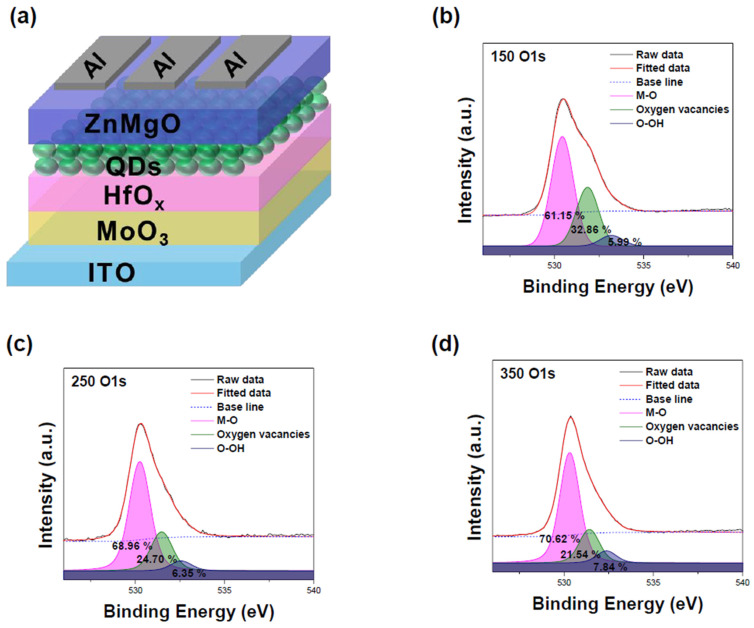
(**a**) Schematic illustration of the device. XPS measurement for O 1s of (**b**) MoO_3_/HfO_x_ (150 °C), (**c**) MoO_3_/HfO_x_ (250 °C), and (**d**) MoO_3_/HfO_x_ (350 °C).

Figure 1a displays the device structure. ITO was used as the anode, MoO_3_ as the hole injection layer (HIL), HfO_x_ as the hole transport layer (HTL), QDs as the emitting layer (EML), ZnMgO as the electron transport layer (ETL), and Al as the cathode. Figure S1a is a cross-section HR-TEM image of the device. The thickness of the HfO_x_ layer was found to be approximately 21 nm. Due to the thin HfO_x_ film, measuring the Hf 4f peak on the ITO substrate via XPS is challenging because it overlaps with the In 4d peak [25]. Therefore, thin films of HfO_x_ were fabricated on a bare Si substrate under the same conditions as device fabrication, with varying annealing temperatures. Figure 1b–d shows the XPS O 1s spectra of the HfO_x_ film at different annealing temperatures. The O 1s peak at ~530.4 eV indicates metal-oxide bonding, while the peak at ~531.5 eV represents oxygen vacancies. The ratio of metal oxide to oxygen vacancies decreases from 1:0.59 at 150 °C to 1:0.31 at 350 °C. Since oxygen vacancies create defect states below the Fermi level in the HfO_x_ bandgap, the annealing temperature influences the defect states of HfO_x_ [26]. Figure S1b–d show the XPS Hf 4f spectra of the HfO_x_ films at different annealing temperatures. The Hf 4f_7/2_ peak is located at 16.9 eV and the Hf 4f_5/2_ peak is at 18.6 eV for all temperatures, consistent with reference values [22]. The XPS Cl 2p spectra of the HfO_x_ film were only measured for the film annealed at 150 °C, as shown in Figure S2d. The presence of residual Cl is due to the incomplete decomposition of HfCl_4_ below 200 °C [24]. These XPS spectra indicate that the annealing temperature of the HfO_x_ films changes the film stoichiometry and the ratio of metal oxide to oxygen vacancies. In particular, the variation in oxygen vacancies is expected to influence the defect states of the HfO_x_ film, thereby affecting hole injection in QLEDs.

**Figure 2:**
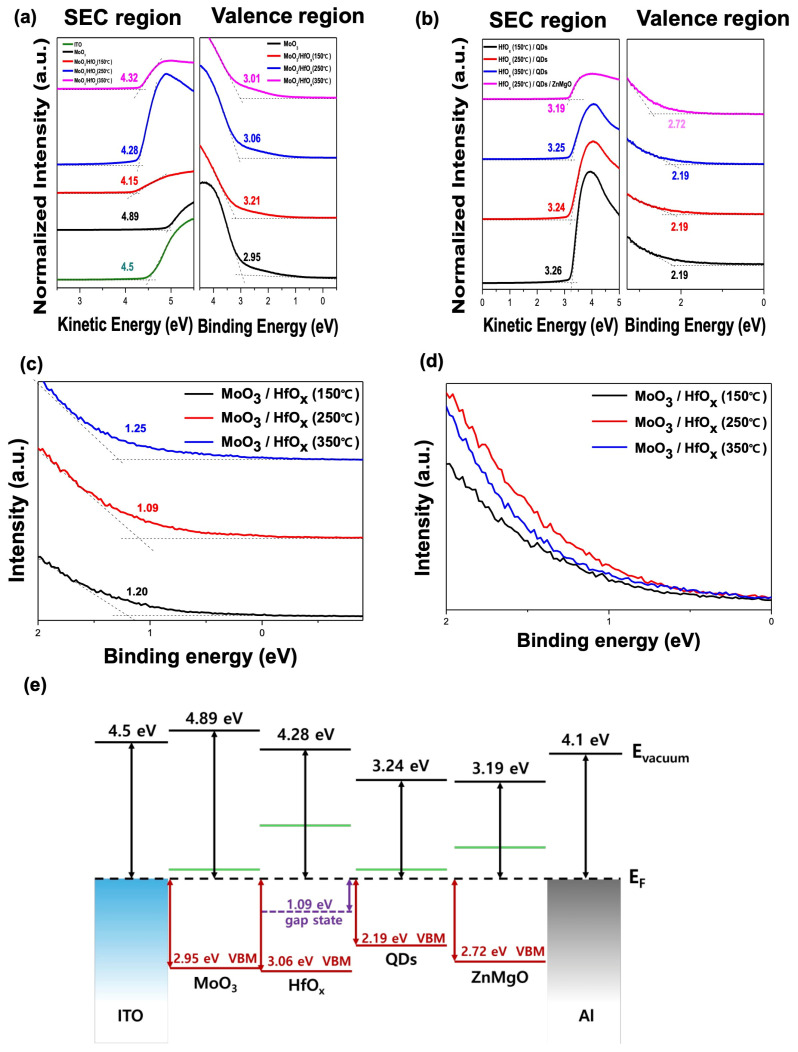
SEC and valence region spectra of (**a**) ITO, ITO/MoO_3_, ITO/MoO_3_/HfO_x_ (150 °C), ITO/MoO_3_/HfO_x_ (250 °C), ITO/MoO_3_/HfO_x_ (350 °C) films, and (**b**) ITO/MoO_3_/HfO_x_ (150 °C)/QDs, ITO/MoO_3_/HfO_x_ (250 °C)/QDs, ITO/MoO_3_/HfO_x_ (350 °C)/QDs, ITO/MoO_3_/HfO_x_ (250 °C)/QDs/ZnMgO films. (**c**) Near valance region of each HfO_x_ film showing the different positions of defect states. (**d**) Near valance region of each HfO_x_ film showing the different intensity (**e**) schematic energy level diagram of QLEDs (250 °C).

Figure 2 shows the UPS measurements and the band structure of the optimized device for the following films: ITO, ITO/MoO_3_, ITO/MoO_3_/HfO_x_ (150 °C), ITO/MoO_3_/HfO_x_ (250 °C), ITO/MoO_3_/HfO_x_ (350 °C), ITO/MoO_3_/HfO_x_ (150 °C)/QDs, ITO/MoO_3_/HfO_x_ (250 °C)/QDs, ITO/MoO_3_/HfO_x_ (350 °C)/QDs, and ITO/MoO_3_/HfO_x_ (250 °C)/QDs/ZnMgO. The UPS-determined work function (WF), valence band maximum (VBM) region, and secondary electron cutoff (SEC) region are shown in Figure 2a,b. Figure 2c presents the near valance region of each HfO_x_ film showing the different position of defect states, which form below the Fermi level due to oxygen vacancies, and vary with the annealing temperature. The defect states of HfO_x_ annealed at 150 °C, 250 °C, and 350 °C are located 1.20 eV, 2.09 eV, and 1.25 eV below the Fermi level, respectively. Figure 2d shows that the UPS intensity near the defect states of HfO_x_ varies with the annealing temperature. The intensity changes with the annealing temperature, with the HfO_x_ film annealed at 250 °C exhibiting the highest intensity, indicating the greatest concentration of defect states. Figure 2e is a schematic band diagram of the device using UPS data from the HfO_x_ film annealed at 250 °C. MoO_3_, when used as an HIL, is known for facilitating hole transport via electron extraction from the CBM due to its deep-lying electronic states [27,28]. The defect states of HfO_x_ are well aligned between the CBM of MoO_3_ and the VBM of the QDs, making it effective for hole injection into the EML. Additionally, HfO_x_ is an insulator with a high CBM—it effectively blocks electrons from the QDs, which helps maintain charge balance [2]. UPS measurements of the device films revealed that the position and concentration of defect states in HfO_x_ shift according to the annealing temperature due to changes in oxygen vacancy concentration. Analysis of the schematic band diagram derived from UPS data illustrates the band structure and defect states of HfO_x_, confirming its advantages as an HTL in QLEDs.

**Figure 3:**
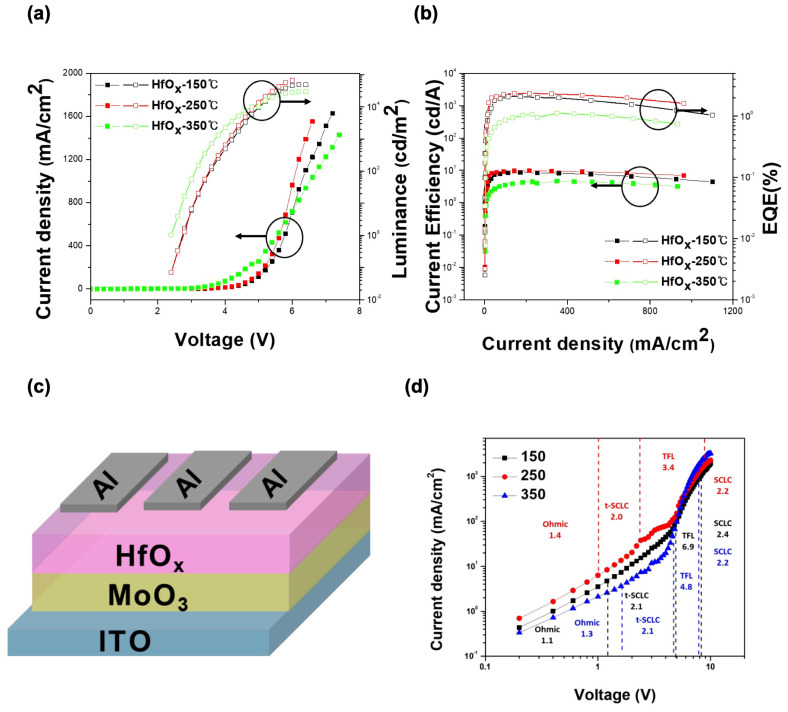
(**a**) J–V–L characteristics and (**b**) current efficiency–current density plot of QLED devices. (**c**) Schematic illustration of hole-only device. (**d**) J–V characteristics of HOD devices.

To confirm the effect of the aforementioned results on QLEDs, we measured the density–voltage–luminance (J–V–L) characteristics of QLEDs with HfO_x_ annealed at different temperatures and density–voltage (J-V) measurements on hole-only devices (HODs) with an ITO/MoO_3_/HfO_x_/Al structure. Figure 3a,b show the luminance, current efficiency, and EQE of each device. Although the turn-on voltage was the same at 2.7 V for all three devices, the device annealed at 250 °C exhibited the highest performance in all aspects, with maximum values of 66,528 cd/m^2^ for luminance, 9.7 cd/A for current efficiency, and 2.4% for EQE, respectively. The structure of the HODs is shown in Figure 3c. Figure 3d shows the current density–voltage (J–V) characteristics of the hole-only device (HOD) at different annealing temperatures of HfO_x_. Four distinct regions are observed: ohmic contact, trap-partially-filled space charge limited currents (T-SCLC), trap-filled-limited (TFL) region, and Child’s law of space charge limited currents (SCLC). The device annealed at 250 °C shows the TFL region at the lowest voltage, indicating the highest hole mobility and the lowest trap concentration [29,30,31]. Figure S2c–e compared the EL intensity peaks of QLEDs with HfO_x_ annealed at different temperatures. The comparison at every 1 V from 3 V to 7 V shows that the EL peak positions are the same under all conditions. These data indicate that the differences in defect states of the HfO_x_ film affect hole injection, leading to variations in device performance.

**Figure 4:**
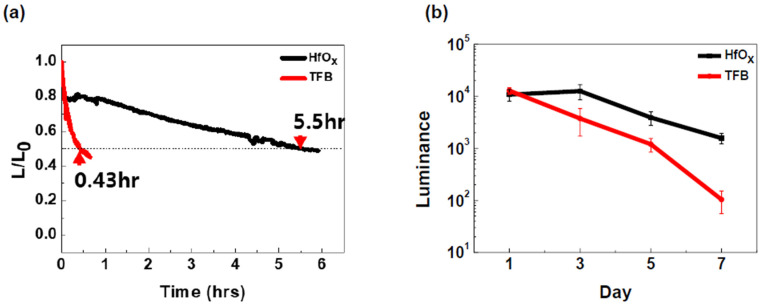
(**a**) Lifetime data of QLED devices using HfO_x_ and TFB for HTL. (**b**) Luminance value measured data every two days of QLED devices using HfO_x_ and TFB for HTL.

All-inorganic QLEDs are known to have superior stability compared to QLEDs using organic materials. Figure 4 shows the stability comparison between QLEDs using TFB as the HIL and other QLEDs. The measured QLEDs were fabricated under the same conditions except for the HTL. Figure 4a shows the lifetime data. The lifetimes of QLEDs using TFB and HfO_x_ as the HTL were measured to be 0.43 h and 5.5 h, respectively. Additionally, Figure S2f shows the evolution of EL spectra during the operational lifetime measurement. These data demonstrate that the device with HfO_x_ exhibited a lifetime approximately 11 times longer, with no significant changes in the EL spectrum until T_50_. Lifetime is a representative measurement to estimate the stability of QLEDs, but it shows degradation occurring over a relatively short period [32]. Figure 4b displays the degradation of QLEDs over a comparatively longer period in an ambient environment. After fabricating the devices, they were stored in ambient conditions, and luminance values were measured at 5 V every two days. Each measurement was taken from five devices, and the results are presented with error bars. It was observed that the luminance of the device using TFB as the HTL decreased more significantly with time compared to the device using HfO_x_. Through these stability data, it was confirmed that the all-inorganic QLED using HfO_x_ as the HTL has superior stability compared to the device using TFB.

## 4. Conclusions

In this study, we developed a highly stable oxide-based HfO_x_ HTL, instead of the conventional organic TFB HTL. XPS and UPS analyses of the HfO_x_ film revealed the presence of oxygen vacancies, which resulted in the formation of defect states in the solution-processed film. Additionally, we observed that the oxygen vacancies concentration and defect states changed with the annealing temperature during the film formation process. Optimizing these characteristics, we successfully fabricated high-stability QLEDs using HfO_x_ as the HTL. The optimized device, achieved by adjusting the annealing temperature of HfO_x_, exhibited maximum values of 66,528 cd/m^2^ for luminance, 9.7 cd/A for current efficiency, and 2.4% for EQE. Additionally, the device exhibited an 11-fold increase in lifetime and greater stability in ambient conditions compared to the TFB-used device. Our results suggest that we have developed a novel HTL, which is crucial for all-inorganic QLED research, offering a useful and simple method for fabricating highly stable QLEDs.

## Data Availability

The authors declare that all data supporting the findings of this study are available within the paper and its Supplementary Information.

## Supplementary Materials

The following supporting information can be downloaded at: https://www.mdpi.com/article/10.3390/ma17194739/s1, Figure S1. (a) TEM Image of QLED device. XPS measurement for Hf 4f of (b) MoO_3_/HfOx (150 °C), (c) MoO_3_/HfOx (250 °C), and (d) MoO_3_/HfOx (350 °C). (e) XPS measurement for Cl 2p of MoO_3_/HfOx (150 °C). Figure S2. (a) The optical band gap of CdSe/ZnS green QDs. (b) The Photoluminescence spectra of CdSe/ZnS green QDs. Normalized EL intensity of devices with HfOx annealed at (c) 150 °C (d) 250 °C, (e) 350 °C. (f) EL intensity spectra during the operational lifetime. Figure S3. Thermal stability of the fabricated all-inorganic QLED devices. Figure S4. The PL intensity variation under different HfOx conditions.
