# Supplementary material for: Improvement of the Stability of Quantum-Dot Light Emitting Diodes Using Inorganic HfOx Hole Transport Layer
Source: Materials (Basel). 2024 Sep 27;17(19):4739. doi: 10.3390/ma17194739 (PMC11477746; doi:10.3390/ma17194739)
Supplement: Supplementary file 1 [file materials-17-04739-s001.zip › materials-3225411-supplementary.pdf]

Supporting Information for:

Improve the stability of quantum-dot light emitting diodes using inorganic HfO_x hole transport layer

Jung Min Yun[a,b] , Min Ho Park[a, b],Yu Bin Kim[a,b], Min Jung Choi[a,b], Seunghwan Kim[c,e],Yeonjin Yi[e], Soohyung Park[c,d], Seong Jun Kang[a,b,*]

[a] Department of Advanced Materials Engineering for Information and Electronics, Kyung Hee University, Yongin 17104, Republic of Korea

[b] Integrated Education Program for Frontier Materials (BK21 Four), Kyung Hee University, Yongin 17104, Republic of Korea

[c] Advanced Analysis and Data Center, Korea Institute of Science and Technology (KIST), Seoul 02792, Republic of Korea

[d] Division of Nanoscience & Technology, KIST School, University of Science and Technology (UST), Seoul 02792, Republic of Korea

[e] Department of Physics, Yonsei University, Seoul 03722, Republic of Korea

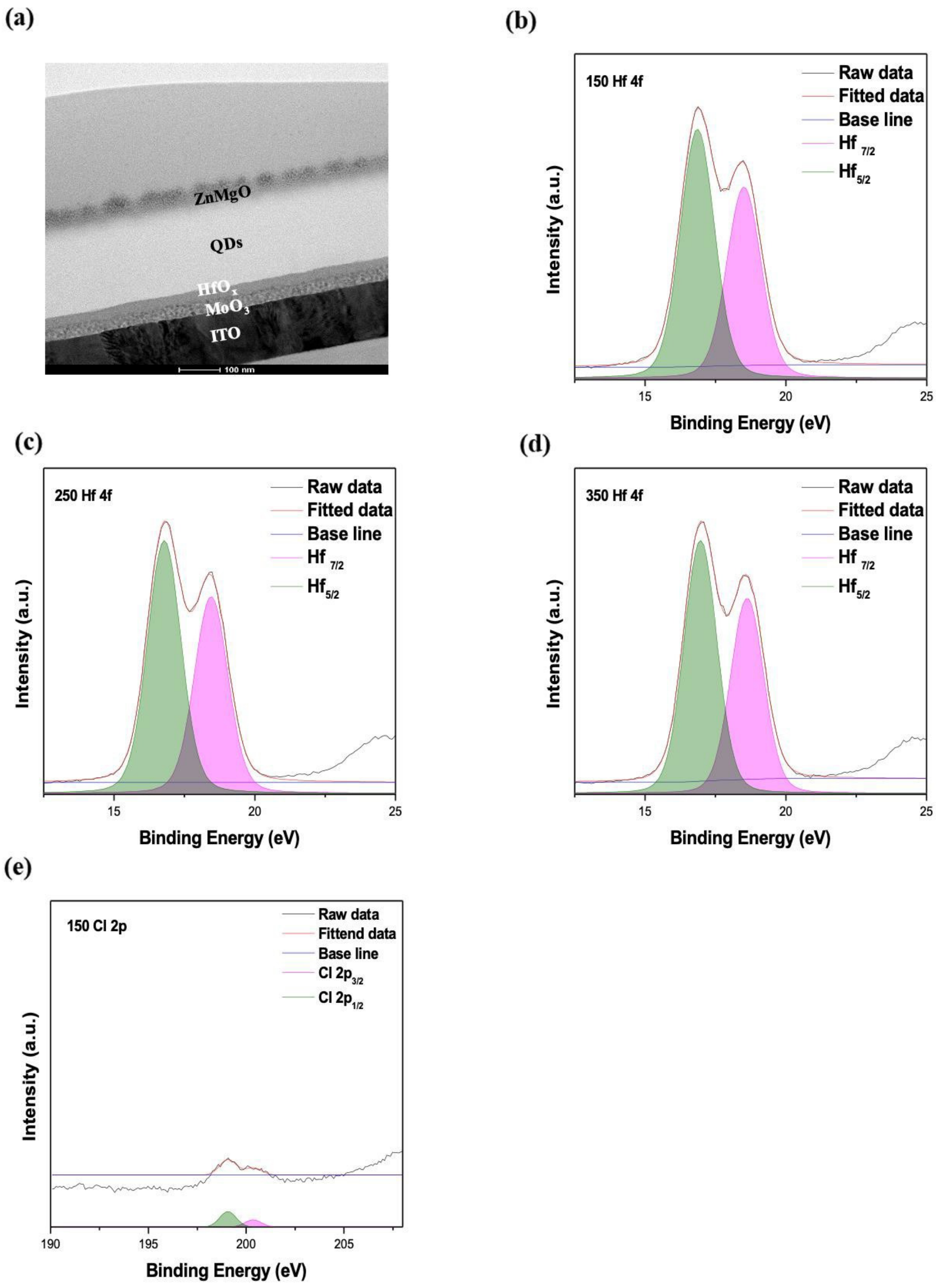

Figure S1. (a) TEM Image of QLED device. XPS measurement for Hf 4f of (b) MoO_3/HfO_x(150°C), (c) MoO_3/HfO_x(250°C), and (d) MoO_3/HfO_x(350°C). (e) XPS measurement for Cl 2p of MoO_3/HfO_x(150°C)

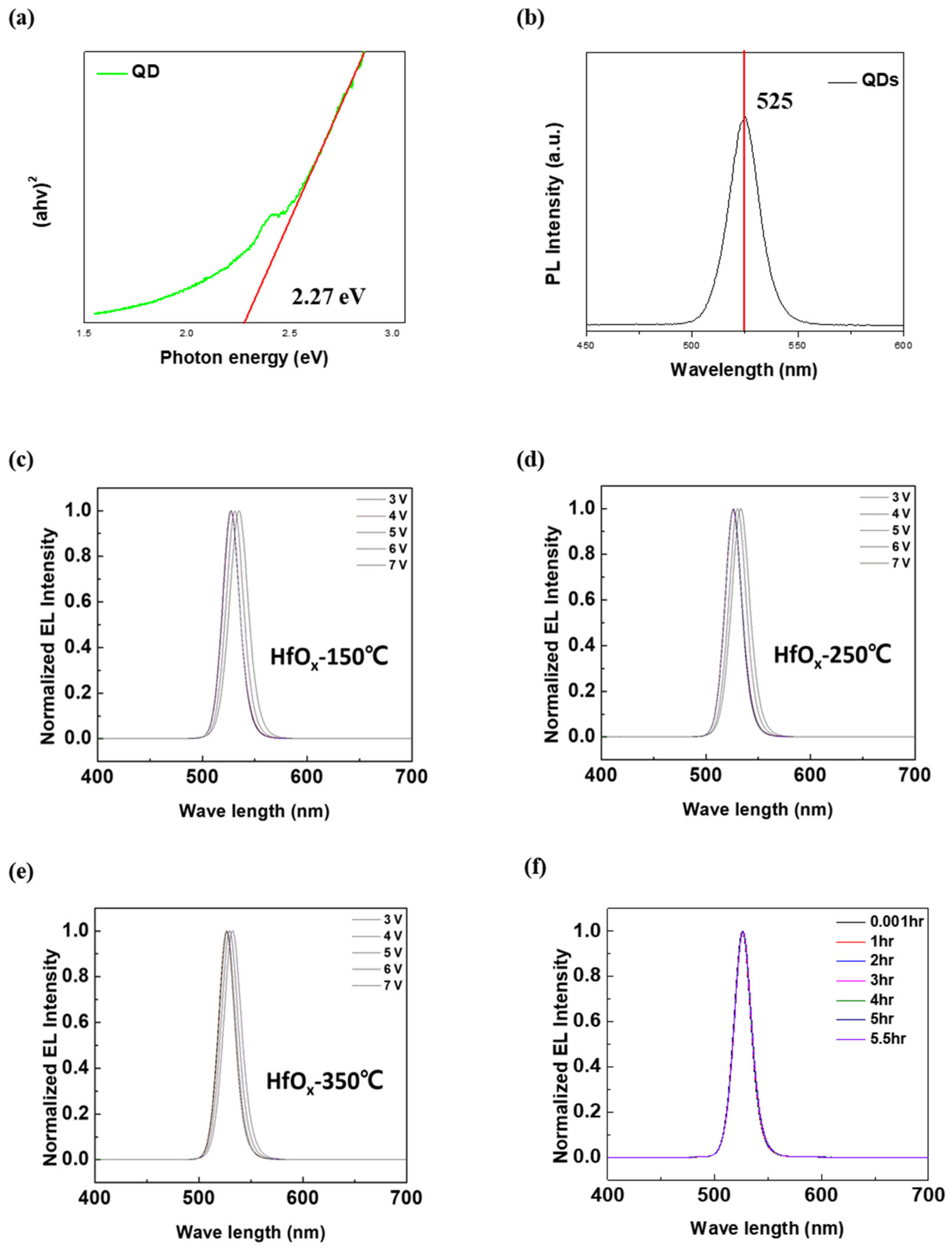

Figure S2. (a) The optical band gap of CdSe/ZnS green QDs. (b) The Photoluminescence spectra of CdSe/ZnS green QDs. Normalized EL intensity of devices with HfOx annealed at (c) 150℃ (d) 250℃, (e) 350℃ . (f) EL intensity spectra during the operational lifetime.

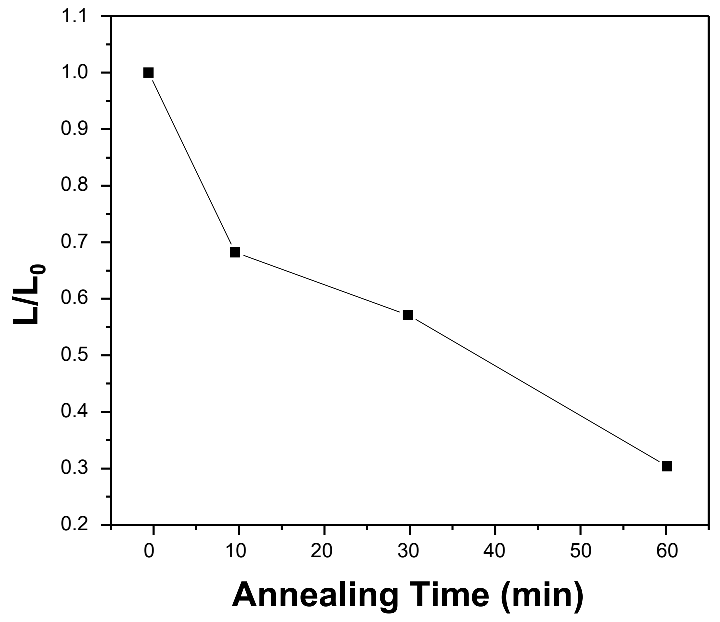

Figure S3. Thermal stability of the fabricated all-inorganic QLED devices.

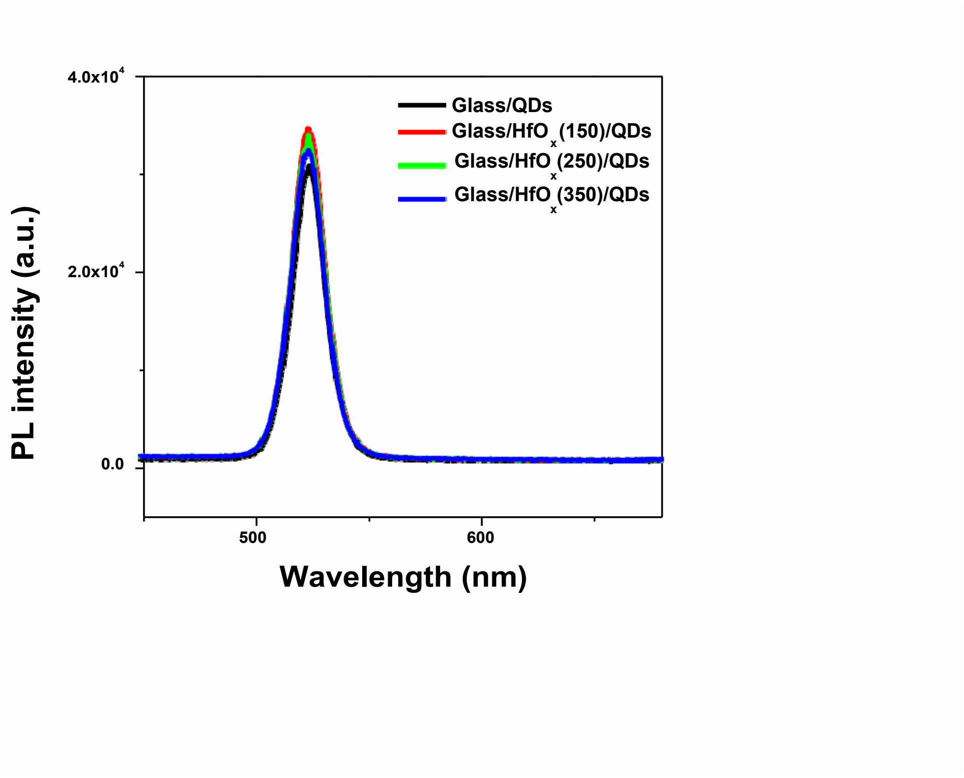

Figure S4. The PL intensity variation under different HfOx conditions.
